# ‘Falling down the rabbit hole’: a thematic analysis of young people’s views on TikTok algorithms and eating disorder content

**DOI:** 10.1186/s40337-025-01505-6

**Published:** 2025-12-20

**Authors:** Tamsin Parnell, Daniel Hunt, Jessica Wilkins, Başak İnce, Helen Sharpe, Ulrike Schmidt, Heike Bartel

**Affiliations:** 1 University of Nottingham, Nottingham, UK; 2 King’s College London, London, UK; 3 University of Edinburgh, Edinburgh, UK

**Keywords:** TikTok, Social media, Eating disorders, Algorithms, ED content, Young people, Qualitative thematic analysis

## Abstract

**Background:**

TikTok’s algorithm is at the centre of its user experience. The platform, which allows users to create and consume short-form content, can enable young people (YP) to feel less alone when experiencing illnesses such as eating disorders (EDs) by encouraging them to build communities around mental health-related content. However, emerging research suggests TikTok’s algorithm might exacerbate ED symptoms by leading YP into spirals of ED content. This paper provides a lived experience perspective on what experiencing high volumes of ED content on TikTok’s For You Page (FYP) can be like for YP.

**Methods:**

We conducted 17 semi-structured interviews in which participants (UK-based, aged 18–25, with experience of disordered eating or an ED) described their experiences of using TikTok. We identified three themes that express how participants interact with ED content on TikTok: (1) View One, See More, (2) Morbid Curiosity, and (3) From Helpful to Unhelpful. Theme 1 describes viewing one unhelpful video and then being exposed to more of this content on the FYP. Theme 2 refers to YPs descriptions of a strong impulse to view ED content when it surfaces on their FYP even if it is felt to be unhelpful. Theme 3 recounts YPs experiences of viewing potentially helpful content (e.g., pro-recovery videos) and then being presented with pro-ED content on the FYP. Through a close linguistic analysis, we examine how participants talk about how they feel when viewing ED content on TikTok.

**Findings:**

Participants reported engaging regularly with ED content on TikTok. Their interactions with ED content show that the personalised nature of TikTok’s algorithm (which is sensitised to how long a user watches a video) can interact with and exacerbate some ED behaviours and psychologies, leading some YP into what they describe as negative echo-chambers of ED content.

**Conclusion:**

Platforms should assume greater responsibility for their algorithms’ roles in intensifying ED symptoms and improve the efficacy of functions that help users control the content they see. A greater awareness of the role of TikTok in exacerbating ED thoughts and behaviours among mental health professionals is also necessary.

**Supplementary Information:**

The online version contains supplementary material available at 10.1186/s40337-025-01505-6.

## Abstract (plain language summary)

This study explored how young people with a current or past eating disorder (ED) describe their experience of viewing ED content on the social media platform TikTok and how this affects their ED and recovery. We interviewed 17 UK-based young people (aged 18–25) with lived experience of an ED. Data showed that the personal experience with the TikTok algorithm – which shows curated content to each user through its ‘For You Page’ – can lead users into what they perceive as ‘negative spirals’ of ED content, increasingly flooding them with ED materials. This can affect, interact with and, in some cases, worsen ED-related thoughts and behaviours of the young people.

**Supplementary Information:**

The online version contains supplementary material available at 10.1186/s40337-025-01505-6.

## Background

Young people’s (YP) use of social media (SM) is ‘almost ubiquitous’ (Yang, Holden and Ariati, 2021: 631). SM allows YP to develop social connections [39] and engage in civic and political life [6]. It enables them to practise identity and relationship management strategies [50] and can support elements of educational and cognitive development [20]. However, [48] warn that SM also offers YP opportunities to engage in risky behaviours outside of parental oversight. As adolescence is a time when well-being shows the most fluctuations [29] and risk-taking is at a peak [47], parents, policymakers and scholars seek to understand the effects of social media use (SMU) on youth mental health [28, 48]. This also includes more research into the addictive potential and psychological and behavioural impacts of TikTok as highlighted by [11].

The porous borders between helpful and unhelpful SM content are particularly salient for YP with EDs and their online engagement with ED-related content. Research into the role of the internet for those affected by EDs has established a positive impact with regards to community building [3, 21, 22, 27], social support and validation [2] and reducing barriers to help-seeking [25]. However, studies have also identified harmful effects of online spaces for people with EDs. Numerous projects have analysed pro-ED websites [38, 42, 46] and pro-ED accounts on SM sites such as Twitter/X [1] and Instagram [19]. These studies have recognised the fine line between helpful and unhelpful or even harmful content in which even spaces that are ostensibly pro-recovery can include unhelpful and potentially harmful content on diet culture, triggering imagery [45], and appearance.

While previous studies have addressed the role of health forums or SM applications like Instagram in exacerbating ED symptoms, recent research has begun to consider ED and ED recovery content on TikTok (see, for example, [23, 24]. TikTok is an algorithm-driven application that, among other things, allows users to consume and create short-form video content [24, 32]. The application has extensive global reach, is popular with YP, and sees high levels of engagement with mental health content [22]. TikTok is therefore ‘emerging as a pivotal tool’ [41] for health information dissemination with attendant concerns about the quality of content and dis/misinformation [15, 51], as well as the potential to exacerbate ED symptoms [23] see below).

Existing studies on TikTok and EDs have typically categorised the type of content found under disorder-specific hashtags, such as #anarecovery and #arfidrecovery [22] and general recovery hashtags, such as #EDrecovery [24]. At the time of writing (April 2025), the #EDrecovery tag is used in over 323,000 TikTok videos. Previous studies (e.g., [13, 24] have identified the prevalence of a TikTok challenge called What I Eat In A Day (WIEIAD), where users record short-form videos documenting their daily meals and snacks in a food-diary format. The genre of WIEIAD videos reflects the public mundanity of much social media content (e.g., Get Ready with Me videos, or GRWM), which focuses on “everyday-ness”. However, WIEIAD videos are given particular significance in the context of ED recovery because they can encourage comparison or promote unhealthy behaviours including rigid monitoring of food and exercise. For example, [13] thematically categorised 100 videos posted to the hashtag #WhatIEatInADay, finding 15 videos in which creators discussed engagement in disordered eating behaviours such as misusing laxatives and skipping meals (2023). In an experimental study, individuals exposed to low calorie WIEIAD videos reported increased social comparison and decreased body dissatisfaction compared to participants exposed to higher calorie WIEIAD videos [14]. In addition to the WIEIAD trend, [24] also identified that some users post weight gain “glow ups” under the hashtag #EDrecovery; in these posts, two contrasting pictures show a body at a very low weight and the same weight-restored body.

What sets TikTok apart from other SM platforms is the centrality of its content selection algorithm, which determines what appears on each user’s For You Page (FYP, a page which shows personalised content to each user). In comparison to other SM platforms such as Instagram or X (formerly Twitter), TikTok places less emphasis on following and subscribing to accounts, leading to a much less explicit representation of the user’s network from which the content they see is drawn; instead, the algorithm directs the flow of content. In other words, although algorithms are used across a wide range of SM, TikTok is the only platform that puts the algorithm at the core of the social experience it offers its users [4].

A recent study by [23] indicates that TikTok’s algorithm may exacerbate eating disorder symptoms. Building on the work of a Wall Street Journal [49] investigation into how TikTok’s algorithm works, the study suggests the algorithm is sensitised to how long a user watches a video for. This means that the longer someone watches a video, the more likely they are to see similar content on their FYP. In the context of EDs, Griffiths et al. imagine a scenario in which a young person engages with, instead of scrolling past, a video that they might find less helpful for their ED because it, among other things, intensifies anxiety or encourages them to compare their appearance unfavourably to another person. This longer view-time increases the likelihood that the user will be exposed by the algorithm to *more* of this unhelpful content on their FYP. In short, a vulnerable user could find themselves in a situation whereby they are repeatedly exposed to ED-related videos because the algorithm has identified this type of content as something with which the user engages, either actively or passively.

As mental health professionals may have a limited understanding of the functionality of the TikTok algorithm and how it might exacerbate symptoms of mental illness [32], it is necessary to develop research in this area that can enrich clinical understandings of how exposure to the TikTok algorithm can affect YP with EDs. Here, we offer lived experience findings that can illustrate how TikTok’s algorithm interacts with ED content to affect YP with EDs. In so doing, we respond to longstanding calls to understand the complexities of online health information beyond reductive concerns with information accuracy and to consider users’ consumptive practices and their nuanced effects [35].

## Methods

### Recruitment and participants

Ethical approval for this study was granted by the University of Nottingham (approval reference code: R2324/022). Two UK ED charities, Beat and First Steps, helped with recruitment via social media (X, Instagram, TikTok), on websites and in newsletters.

Participants had to be aged between 16 and 25. Parental agreement procedures were in place for participants below 18 years old, though the data reported in this paper includes only participants aged 18 and over. Participants had to live in the UK, have experienced an ED or disordered eating (with or without formal diagnosis), and have posted or viewed ED content on social media. Recruitment advertisements explicitly encouraged participation from individuals usually under-represented in existing eating disorder narratives including those who live rurally, identify as LGBTQ+, as ethnically or culturally diverse, and/or as experiencing resource insecurity.

Potential participants completed an expression of interest (EOI) form and in response were sent study information and consent forms. Once they had given their written informed consent, an online interview was organised, and the research questions were shared (see Appendix 1 for interview questions). In total, there were 67 responses to the EOI form with 30 participants responding to a follow-up email and consenting to an online interview, which was held and recorded via Microsoft Teams. As the interviews were semi-structured, a broad interview guide was followed, although there was reasonable flexibility in response to participants’ answers. Interview transcripts were stored securely in a private Microsoft Teams channel hosted on a secured university server, and accessible only to the research team.

As the interviews addressed potentially emotionally salient topics, we ensured that all interviewees received a signposting information sheet after participation which outlined where the participant could go for support if needed. Participants were regularly offered time to pause and reflect and were reminded repeatedly that they could choose to answer as little or as much as they liked. It was made clear that participants did not have to respond to questions they did not want to answer and could stop the interview at any time without repercussions.

After the interview, each participant received a £15 voucher. To reduce the potential for financial compensation to lead to fraudulent participation of people who did not meet our inclusion criteria (a problem discussed in relation to ED research in [13], we asked participants to put their cameras on at the start of the interview. This, we hoped, would at least deter those who did not meet the age criterion. A second reason for asking for visual confirmation of the participant was to ensure that the same participant was not taking part in the interviews more than once; previous qualitative interview studies (e.g., [40] have experienced this problem with participants who have remained off-camera and participated more than once to receive financial compensation several times. This is particularly foregrounded as an issue when studies rely on snowball sampling [44]; while we had one participant take part who was recommended by a friend, we were able to ascertain via camera that this was not the same person.

While the interviews focused on participants’ experiences of engaging with ED content on social media broadly, here we present data from 17 of these interviews in which participants spoke specifically about their TikTok use and its effects on them. We present responses from a subsample of 17 interviews rather than the 30 people interviewed not because the other 13 respondents provided systematically different interpretations, but rather because these 17 participants were the only ones who explicitly discussed the effects of *TikTok* on their mental health. Other participants typically mentioned other platforms (e.g., Tumblr, X, Reddit, YouTube) and their responses, while insightful, were consequently out of scope for the focus of this paper.

Participants were not asked to disclose their specific type of disordered eating or ED, but as open questions encouraged them to share as much or as little as they felt comfortable sharing, many did disclose an ED diagnosis. All participants said that they used apps such as TikTok daily, although we did not ask about the time spent on social media. Table 1 presents the characteristics of the participants. We would like to draw attention here to the fact that our subsample largely consists of young women with experience of anorexia; this should be considered alongside the interpretation of our findings.

**Table 1:** Participant characteristics

Participant ID	Gender	Lens(es)	Diagnosis disclosed
1	Female	Living rurally	Anorexia nervosa
3	Female	LGBTQ+	Anorexia nervosa
6	Female	Resource insecurity	Disordered eating (self-diagnosis of orthorexia)
7	Female	Living rurally	Anorexia nervosa and bulimia
8	Female	Cultural diversity	Atypical anorexia and then anorexia nervosa
9	Female	Resource insecurity	Anorexia nervosa
10	Female	Ethnic diversity	Disordered eating
11	Female	Living rurally	Disordered eating
12	Female	LGBTQ+	Disordered eating (self-diagnosis of bulimia)
15	Female	LGBTQ+, resource insecurity	Anorexia nervosa
16	Female	LGBTQ+	Disordered eating
19	Female	Does not identify with our lenses	Anorexia (with bulimia)
20	Female	Ethnic diversity	Anorexia nervosa
21	Female	LGBTQ+	Anorexia nervosa
22	Female	LGBTQ+.	Bulimia
23	Female	Does not identify with our lenses	Disordered eating
27	Female	Does not identify with our lenses	Anorexia nervosa

### Analytical process

The analytical process we adopt in this project is thematic analysis [7, 8]. In line with [7], we understand a theme to be a patterned response in our dataset that does not have to be the most quantitatively important pattern, but which contributes to an important aspect of meaning-making in the data. The data analysis process was inductive; we did not ask specific questions about TikTok algorithms and ED-related content during the interviews but rather found this was a salient topic across the interview responses and was therefore worthy of greater attention.

We followed the six-step process for reflexive TA that is briefly outlined in [9]: familiarisation, coding, generating initial themes, reviewing and developing themes, refining, defining and naming themes, and writing up. First, TP transcribed the interviews from audio and video recordings. Then, in line with the familiarisation stage, three authors (TP, DH, HB) read the interview transcripts independently several times. As coding is subjective at its core [9], the three authors initially identified codes independently. The three authors adopted a manual approach to coding, using pen, paper, and different coloured highlighters to identify recurrent patterns of meaning rather than software like NVivo. Next, the authors came together to discuss their findings and discovered overlaps, especially across the following codes: representations of agency, depictions of the algorithm as sentient or powerful, and consumption of ED content as compulsive (generating initial themes). Together, through extensive discussion, the authors generated the three “key” higher-level themes that brought together the codes by reflecting *how* the participants described their interaction with a powerful algorithm and a compulsion to view ED content through the lens of (lack of) user agency (reviewing and developing; naming themes). The authors then selected extracts that reflected each of the key themes most closely. All three authors analysed the extracts independently to identify key linguistic features of interest related to agency, disempowerment, and depictions of the algorithm. The authors then came together again to share their findings and agree on a mutual interpretation of each of the extracts that would lend itself to an overall argument (refining, defining, linguistic analysis).

During these meetings these authors also reflected on their own experiences as social media users as well as their previous research with individuals with EDs and on online contexts and how these may have shaped their responses to the participants’ data. The method adopted therefore aligns with *reflexive* thematic analysis (TA), which, according to Braun and Clarke [9]: 330), understands the researcher’s subjectivity as ‘analytic *resource’* (italics in original). In other words, in line with a constructivist interpretation, the analysis presented below is co-constructed – that is, influenced by participant responses but also by what the authors bring together as a research team comprised of, *inter alia*, linguists, experts in health humanities, and psychologists. The focus on the language used to convey agency, for example, is influenced by a curiosity about language and health, while the intersection between mental health and technology reflects a broader interest in health humanities and psychological interactions with social media.

The Standards for Reporting Qualitative Research (SRQR) also recognise the importance of reflecting on the potential or actual effects of researcher characteristics, biases and beliefs to interact with RQs, approach, methods and results. We discovered through discussion that our previous scholarly and personal experience with disordered eating allowed us to recognise the vulnerable and emotional nature of the topic, enabling us to maintain sensitivity, patience and empathy during the interviews. We also sought to support one another post-interview by meeting to discuss any feelings of distress among our research team. In what follows, we present our findings from the analysis of the aforementioned codes and themes in line with the recommendations of the Standards for Reporting Qualitative Research [36]; Supplementary Table 1).

## Results

Throughout the interviews, there was one type of content that YP positioned as “helpful”^1^ on TikTok: images or videos of people with EDs having full and rich lives outside of their ED. Three types of content on TikTok were positioned by YP as “unhelpful” with regards to their disordered eating and ED. The “unhelpful” content was: (1) What I Eat In A Day challenges, (2) before and after pictures that visualised weight gain, and (3) gym and fitness content posted by ostensibly recovery-oriented accounts. Our thematic analysis delineated three dominant themes that account for participants’ encounters with this type of content, and which seek to capture both participants’ immediate experiences and the longer-term effects of consuming this content: ‘View One, See More’, ‘Morbid Curiosity’ and ‘From Helpful to Unhelpful’.

### Theme 1: view One, see more

View One, See More, refers to a pattern in which participants describe watching a single video and then being consistently exposed to more of that content, making it hard to escape from. The emphasis in this theme is on users’ lack of control to stop the perceived activities of the algorithm.

During the interview, we asked participants how social media could be improved for YP with ED. Participant 1 responded with reference to TikTok.

#### Extract 1 (P 1)

*TikTok*,* you seem to*,* you know*,* you watch one video that might come up because*,* you know*,* you just watch it. You know you shouldn’t*,* but you do. And then you just get more and more and more. And it just sort of feeds itself and*,* you know*,* you can try and say not interested*,* but it- it’s- just sort of seems to be working against you sometimes.* (P1)

Here the participant emphasises a passive consumption of ED content – she ‘just watches’ one video that ‘come[s] up’ and then ‘gets more and more and more’. In contrast, the TikTok algorithm is depicted as highly agentive, consumptive (‘it just sort of feeds itself’) and hostile. The participant’s perceived lack of agency is highlighted in the verb ‘try’ (‘you can try and say not interested’) which suggests that she believes there to be limited options for curating the content she is exposed to;^2^ this lack of agency juxtaposes with the more dynamic, personifying action of the algorithm as ‘working against you’. Throughout the extract, the participant uses the generic pronoun ‘you’ to express the experiences she describes, presenting this as a general experience that recurs for multiple people rather than an isolated incident. Overall, there is a distinct sense of lack of user control over the algorithm, with the algorithm itself personified as an entity that ‘feeds itself’.

(Lack of) control is also a central topic in Extract 2, spoken by Participant 3.

#### Extract 2 (P 3)

*Oh yeah*,* like*,* TikTok. Like*,* I scroll on there*,* but I don’t post. But you never*,* you don’t really have much control over what you see on TikTok*,* which is difficult. That is*,* I do think that is difficult. And I think there’s a lot*,* like*,* because you never really know*,* like*,* if you interact with one post doing something*,* it might send more than [that]. Yeah*,* that’s definitely*,* like*,* it’s a social media that I do try and not go on as much because I can’t control it as much. Like Instagram*,* you know*,* you can control who you follow.* (P3)

In this second extract, the algorithm is depicted as unknowable or unclear – ‘you never really know…it might send more’ – which suggests that, for this participant, attempts to push back against or “game” the algorithm (that is, deliberately alter the content that the algorithm shows) might be ineffective because, she believes, users are not fully aware of how the algorithm might respond. There is a passivity to the participant’s consumption of ED content – ‘I scroll on there’ – that implies she believes users do not necessarily have to be active engagers with ED content to see this on their FYP. Equally, the personifying verb ‘send more’ suggests a very deliberate or conscious act on behalf of the algorithm, again highlighting technological agency in contrast to the user’s expression of disempowerment. Participant 3 also emphasises that they ‘try not to go on as much’ – ‘try’ suggests that the participant, despite her best efforts, does not or cannot avoid the platform altogether.

Extract 3 from Participant 9 highlights ‘easy access’ to ED content on TikTok and recognises a tension between wanting to recover and wanting to stay with the ED:

#### Extract 3 (P 9)

*Yeah. It’s so hard because I think a lot depends on the mental space that you’re in as well*,* because for me sometimes*,* like*,* I’ll be scrolling through and I’ll be like*,* oh my goodness*,* I’m just not- I’m not working hard enough for my recovery*,* like from a disordered eating*,* like*,* perspective. I’m just not working hard enough. My recovery…like they’re doing so much better than me. Then the other part of me is like*,* oh*,* God*,* they’re doing*,* they’re doing so well. Like*,* I don’t want to be like that. I want to just stay in my old unhealthy ways. And then I end up falling down this rabbit hole of looking at these awful*,* like accounts on TikTok. And it’s just such easy access. That’s the real issue for me… is like such easy access to it. You can just see all these people’s*,* like*,* What I Eat In A Days and it’s all just like a banana for breakfast.* (P9)

This user engages in social comparison even for positive recovery behaviours. The metaphor of involuntarily ‘falling down this rabbit hole’ elucidates a perceived lack of agency in the process of going from viewing one or two videos to looking at the accounts behind the videos and presumably watching their other content. This process is linked to ‘easy access’, which places some responsibility on the TikTok platform for making it easy to find and become further exposed to more ED content on the FYP. Meanwhile, TikTok as a platform is implicitly given an agentive role in providing ‘easy access’ to the ED content.

Overall, theme 1 highlights the perception of easy-to-access ED content on TikTok. Participants suggest watching one distressing video can lead to ‘falling down [a] rabbit hole’ of viewing more and feeling like the algorithm is ‘feeding itself’ on their insecurities. Interviewees express lack of agency and do not suggest that they are responsible for ending up in spirals of ED content, using metaphors of involuntarily ‘falling’ that contrast against personifying the algorithm as actively working against them; while the user’s agency is backgrounded, the algorithm is depicted as powerful and uncontrollable.

### Theme 2: ‘Morbid curiosity’

Theme 2, ‘Morbid Curiosity’ (termed by one participant), refers to a scenario in which YP describe knowing how the TikTok algorithm works but having a “morbid curiosity” to view (potentially “unhelpful”) ED content because of their ED. When they *do* watch this content, they state, more of it comes up on their FYP, causing a negative effect. There is also one situation in which the participant describes deliberately seeking out ED content they find distressing to maintain ED thoughts and behaviours. In this theme, the emphasis is on the tension between wanting and not wanting to view the pro-ED content that the algorithm generates. Theme 2 highlights how the ‘easy access’ to ED content on TikTok (extract 3) interacts with a temptation or compulsion to view.

Participant 16 describes a tension between a ‘tempt[ation]’ to click on the account that posted ED content that ‘came up on [her] for you page’ and an internal ‘disappoint[ment]’ in herself for viewing it:

#### Extract 4 (P16)

*And like I don’t know*,* it’s a very strange one because like I… there is a feature. There’s a tool on TikTok which is like you can hold down and say not interested. And I have done that. I don’t*,* but like I can’t lie and say that there wasn’t part of me that went*,* what the hell? And I clicked on the account and as much as it*,* like*,* disappoints me like I…I was always tempted*,* whenever it came up on my For You Page*,* I always wanted to look at the account*,* like*,* as much*,* and like*,* I hated myself for it. But I would. And then I would maybe [click] not interested. But I never sought it out. That’s the thing that really like*,* yeah*,* was strange for me.* (P16).

In this extract, the participant repeatedly describes the experience of a compulsion to consume ED content by clicking on the account behind the video as ‘strange’, suggesting a feeling of disorientation at the tension between not wanting to view the content and the feeling of temptation to look further into it. The words ‘disappoints’ and ‘hated’ point to a strong emotional reaction to the temptation to view, suggesting a degree of self-blame for following through on the compulsion, even though the participant clearly says that she has tried to avoid the videos by ‘hold[ing] down and say[ing] not interested’. That the participant references saying ‘not interested’ echoes Participant 1 in suggesting that some YP say they do try to implement strategies to avoid “unhelpful” content. However, here, because the user views the accounts behind the posts (thereby signalling a degree of interest in the content), attempts at recovery by clicking ‘not interested’ could be thwarted. In other words, we suggest that clicking ‘not interested’ might not have been effective this time because it was combined with an indicator of interest in these types of posts (the viewing of the accounts behind the posts). Overall, the extract suggests that although users are often advised societally that they should curate feeds or use buttons like ‘not interested’, it can be challenging even to be presented with this choice in the first place.

In Extract 5, Participant 8 describes a process of ’giving in’ to a compulsion to view ED content:

#### Extract 5 (P 8)

*I know how the algorithm works and I still get sucked into their like sick pics and all that kind of thing. And I know that the more I engage with them*,* the more it’s going to show me. But it’s just so hard to scroll past all the like*,* What I Eat In A Day and like all that kind of thing. It started showing me weight loss ones recently*,* which is like so opposite of… it’s either recovery or weight loss now and it’s ridiculous. And I know that it’s ridiculous*,* but it’s still so kind of compelling that I can’t help myself.* (P8)

This participant claims explicit knowledge of how the TikTok algorithm works through the repetition of ‘I know’ and the understanding that ‘the more I engage with them, the more it’s going to show me’. This foregrounding of knowledge strikingly juxtaposes with the sentiments of ‘I can’t help myself’, and being ‘sucked in’ by ‘compelling’ content provision, all of which highlight disempowerment in the face of the potent combination of technology and disorder. Participant 8 suggests that the algorithm does not distinguish between ‘recovery’ and ‘weight loss’ content (‘it’s either recovery or weight loss now’), indicating that the participant believes there is scope for the algorithm to “get it wrong” and show exactly the opposite of what the user is looking for. More explicitly, though, this extract suggests that even thinking one knows how the algorithm works still does not result in a feeling of agency for the user(s), as they may be unable to help themselves in the face of content that they find compelling.

Participant 15 explicitly uses the term ‘morbid curiosity’ to describe a process in which ‘your head will just make you keep seeking out that content even though you know it’s going to be tougher’:

#### Extract 6 (P 15)

*And on TikTok*,* I know you can’t mute*,* but you can*,* like*,* suggest it show posts like this less or something. But*,* like*,* I think like I said*,* because of the algorithms*,* it is really hard to kind of avoid that. Because I think as well there’s also this morbid curiosity*,* it’s like sometimes your head will just make you keep seeking out that content even though you know it’s going to be tougher. I think that that’s where that sort of curiosity comes into it*,* where you like*,* you almost want to seek it out so that you end up feeling worse*,* which makes no sense. What we’re doing just sort of doesn’t make sense*,* I think. Yeah. I definitely need to try to*,* like*,* mute and block things that I don’t want to see. Like when I’m in a healthy sort of state of mind.* (P15)

This extract exudes a sense of self-awareness of the paradox of knowingly consuming harmful content. It is underpinned by a duality in which the participant’s ‘head’ autonomously seeks out the content whereas the ‘healthy state of mind’ desires not to see the content. Like in Extract 5, the participant highlights a range of strategies they could use to ‘avoid’ ED content – ‘muting’, ‘blocking’ and expressing that they would like to ‘see posts like this less’ (the button others call ‘not interested’). However, she suggests these might be ineffective in the face of an eating disorder or mental ill-health generally – she says that she ‘definitely need[s] to try to’ implement these strategies but implies that this is only possible ‘when [she’s] in a healthy sort of state of mind’. Again, then, the question of whether an individual must be in a particular mindset at any given point to be able to push back against the ’morbid curiosity’ to view is raised. If this is the case, the strategies available to YP on TikTok (blocking, muting, expressing their lack of interest in a post) may be less effective for those who are most vulnerable, or at the peak of their ED.

Extract 6 also introduces the concept of deliberately seeking out disordered eating content in order to maintain an ED. Similarly, in Extract 7 Participant 7 describes being compelled by ‘the toxic part’ of her brain to go ‘searching’ and ‘look for photos’ where other people are ‘underweight’:

#### Extract 7 (P7)

*Then the toxic part of my brain then goes*,* like*,* searching*,* because then I’ll click on their profile and*,* like*,* look for photos when they’re underweight because I’m like… which obviously I don’t want to do*,* but you just… You just can’t help yourself sometimes. (P7).*

Extract 7 displays a similar duality, causing a confrontation between the part of the brain viewed as ‘toxic’ and the part of the brain that wants to be healthy. The underlying suggestion is that, for this person, TikTok exaggerates the rift between the self that wants to get better and the ‘toxic’ side of the self that wants to continue looking for photos of underweight bodies, with the latter often gaining the upper hand. In this way, the extract implies that there is a particular and potentially harmful interdependence between what the user brings and what the algorithm offers, and the extract helps to explain users’ continued participation on a platform that, at least in part, they identify as harmful.

Overall, Themes 1 and 2 present users as fighting against both the algorithm and the existing compulsion to engage with content that they know will be deleterious. In other words, the salient representation is of an algorithm that selects harmful content that the YP know they will be ‘tempted’, ‘curious’ or ‘compelled’ to engage with. Here, the algorithm is again imbued with agency and power while the user lacks the agency to control the content they view, either due to the algorithm itself or a compulsion to view *more* content once it is initially displayed.

### Theme 3: from helpful to unhelpful

The final theme, From Helpful to Unhelpful, encapsulates scenarios in which there is a trajectory from more positively-oriented or neutral content towards explicitly “unhelpful” content. It frames engagement with ED content as more dependent on TikTok algorithms than the individual viewing the content, either because the algorithm does not distinguish between what is pro-recovery content and what is not, or because it encourages viral challenges that YP explicitly evaluate as “unhelpful” for their recovery (e.g., What I Eat In A Day). This trajectory is illustrated in Extracts 8 and 9 below:

#### Extract 8 (P 6)

*It’s the algorithm*,* so*,* like*,* once you like a video*,* you get more of them. So*,* I think I might have liked someone’s eating disorder recovery video. And then now I get ones that are just related to disordered eating*,* and*,* like*,* I just can’t stop them from*,* like*,* coming up on my page.* (P6)

#### Extract 9 (P 21)

*And even if it’s*,* you know*,* it’s harmful and you know you don’t want to look at it*,* sometimes it gets accidentally put up for you*,* or sometimes it’s something that they want to look at it*,* and it’s knowing it’s out there.* (P21)

Extract 8 exudes some similarity to the View One, See More theme in that it suggests that once you engage with one piece of content, you will be exposed to more of it. However, it also implies that the algorithm cannot distinguish between what is pro-recovery and what is pro-ED content; someone who wants to view positively oriented videos may well be exposed to more harmful content even if they have not searched for it. This suggests the TikTok algorithm could need to be fine-tuned to capture vital differences between pro- and anti-recovery content rather than assuming all ED-related content is the same. Notably, Extract 8 also expresses a sense of helplessness – ‘I just can’t stop them’ - which indicates a potential need for improved efficacy of embedded functions that allow users to curate their feed (e.g., the ‘not interested’ button).

In Extract 9, the participant presents a variety of options for how YP might come across ED content on TikTok – it might get ‘accidentally put up for you’ (an expression which again suggests the indifference of the TikTok algorithm as an instrument for content curation) or a participant might go looking for it. Here, TikTok is perceived as the source of the easy access to the content that facilitates the compulsion to view – ‘it’s knowing it’s out there’.

Whereas participant accounts in Theme 1 construct TikTok’s content curation algorithm as hostile and beyond their control, Theme 3 presents it simply as an indifferent instrument that is not consistently able to distinguish between pro-ED and pro-recovery content. Nevertheless, the upshot for the user – exposure to content that they experience as harmful – is the same. Consequently, it raises questions about the need for a more nuanced algorithm that can recognise differences in content so that it does not group together all ED or weight-loss related videos and offer them to potentially vulnerable users.

## Discussion

**Fig. 1:**
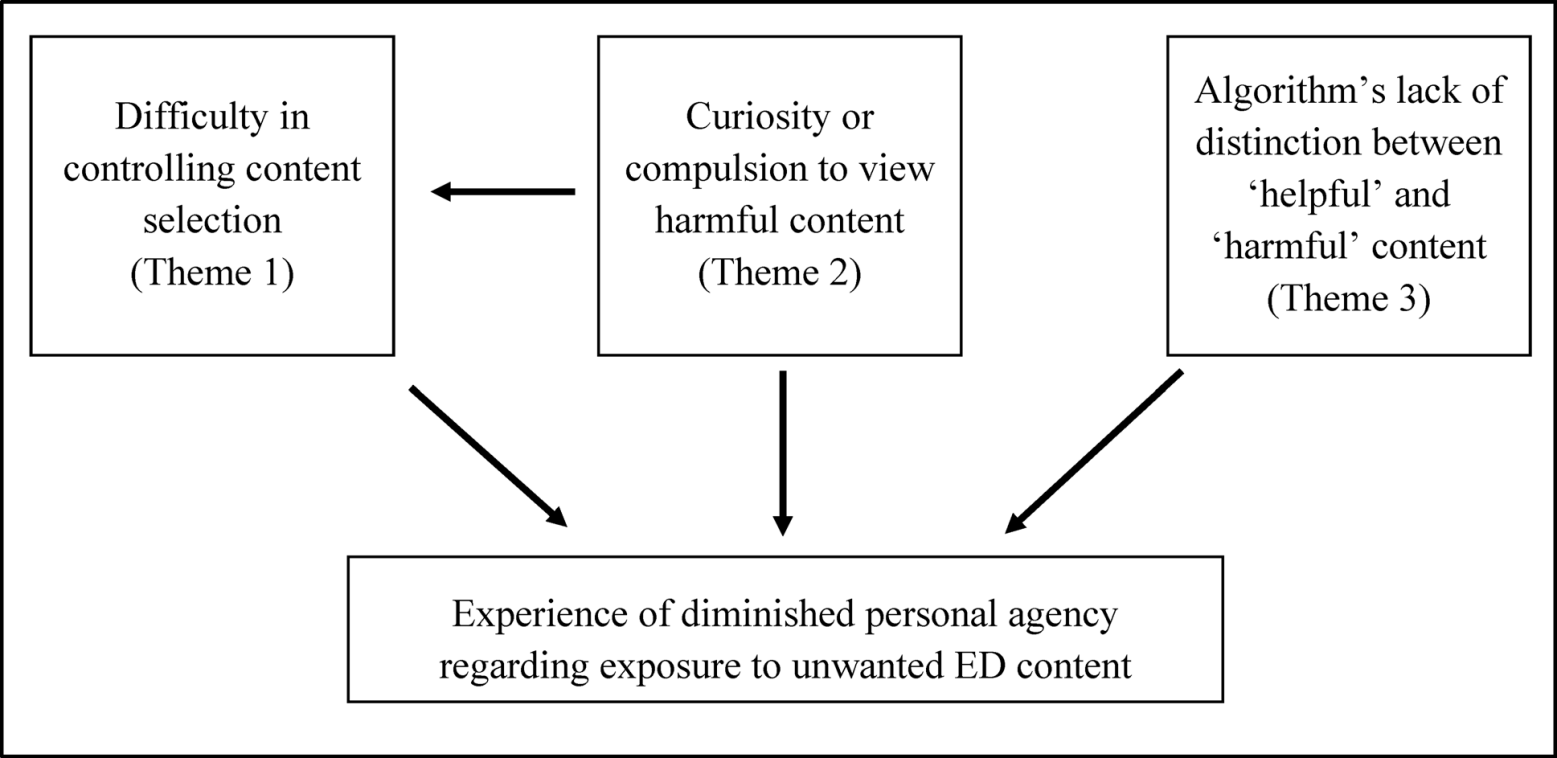
Conceptual diagram illustrating the relationship between the study’s three themes, YPs’ perceived agency, and their consumption of harmful content

This qualitative study sought to illuminate how YP with experience of EDs perceive the viewing of ED content on TikTok. The interviewees express varying degrees of powerlessness with regards to ED content on TikTok and their vulnerability as a consequence of this. This is, in all extracts, related to the function of the platform’s algorithm. The participants’ feelings of powerless range from being exposed to harmful content without wanting to see it (Theme 3, From Helpful to Unhelpful), to not being able to control the constant flow of content that is initiated even if they have only engaged with it once or twice (Theme 1, View One, See More). Even for participants who describe more deliberate engagement with pro-ED videos, they still present themselves as powerless to subsequently shift out of the echo chamber of ED content that the algorithm creates for them (Theme 2, Morbid Curiosity). These themes and the relationship between them and participants’ experiences of reduced personal agency are summarised in Fig. 1.

The way in which users articulate their relationship to TikTok’s algorithm reveals their perception of its particular potency and potential for harm; the participants express that they engage with TikTok in full knowledge that the algorithm will use even a one-time interest in ED-content and ‘feed off it’. This means that users often feel they have limited influence over the content, and that content delivery can easily become unmoderated or overwhelming. This can be regarding the quantity of content, i.e. the algorithm provides much more of the same content than initially asked for, or regarding the quality of the content, i.e. the algorithm moves from presenting ED-related content that is initially helpful to that which is unhelpful or even harmful. A combination of both is also possible. YP may also experience a more conventional peer pressure to participate in a media environment that is highly popular among their demographic, meaning that switching off from TikTok altogether is not perceived as a viable option.

The different forms of personification attributed to the algorithm are striking in the extracts. They highlight the agency that users attribute to it as an entity that takes over and gains more and more control while the user becomes increasingly powerless, trapped and vulnerable – they have ‘fallen down the rabbit hole’, as one participant puts it. In addition, the metaphor of ‘feeding off’ something or someone, like a leech, highlights both the difficulty of shaking off this entity, and the notion of becoming weaker while it grows stronger. Finally, the expression ‘you can try and say not interested, but it – it’s […] working against you’ resonates uncomfortably with notions of physical and emotional violation – the user’s ‘not interested’, requesting a stop of activities, is neither registered nor respected. It is the complexity of this ‘relationship’ with the algorithm that needs to be considered when evaluating the feelings expressed by YP with EDs around their engagement with this highly popular platform that plays such an important part in the lives of their peers. Participants express their engagement with ED-content on TikTok as something ‘they shouldn’t do’, something they recognise as not good for them and potentially harmful, yet also as something they feel strongly attracted to. They direct resulting feelings of guilt or blame about this engagement at themselves, and not directly at the platform. In addition, close analysis shows that users express the dynamic of these feelings not as predominantly caused by their interest in online ED-content per se but regarding their engagement with the algorithm – in what they understand to be their full knowledge of its workings. This knowledge does not make them less vulnerable to its unhelpful mechanisms, though, especially when the socio-cultural role as well as information and entertainment value of this platform is so high among YP [32].

Our research also suggests that some of the limited strategies YP have available to them to avoid unhelpful content on TikTok, such as the “not interested” button, may be less effective for those who are in the most vulnerable state of their ED, because ‘morbid curiosity’ leads them to watch the videos that appear on their FYP, click through to the accounts posting this content, or go actively searching for photos that they find disturbing. The YP interviewed for this study suggested they had to be in a particular mindset to be able to use some of the functions of the app available to them (e.g. muting, clicking “not interested”) – a mindset that enables them to overcome the temptation to view content they find unhelpful or harmful. Similarly, other YP indicated that the TikTok algorithm was in some ways a blunt, indifferent instrument that could not distinguish between pro-recovery and pro-ED content and therefore showed potentially unhelpful content to those who did not search for it. Together, these findings highlight both the necessity of adding nuances to the TikTok algorithm so that it can distinguish between helpful and potentially harmful content and improving the functionality of existing measures so that it can support vulnerable users.

### Strengths and limitations

This study has some limitations that are important to consider: namely, that all the participants whose extracts are analysed identified as female and typically had experience of AN rather than other ED diagnoses. This was not a deliberate sampling decision, but we hypothesise that it might reflect greater taboo around eating difficulties among boys and men [30] and also the characteristics of those engaging with our partner organisations (Beat; First Steps) although we have not verified this.

Future qualitative research on the effect of TikTok algorithms and pro-ED content on those with lived experience must consider how different diagnoses might interact with the algorithm and pro-ED content in different ways, to produce different types of lived experience. It is critically important to also engage boys and young men in this research; while we did interview young men, none of the men spoke about TikTok. In retrospect, asking specifically about image-based platforms like TikTok (which appear to be more harmful to body image than text-based platforms [31] would have provided greater insight from a more balanced gender perspective.

The wider study from which the interview data reported in this paper are taken was developed to explore broader experiences of digital technologies rather than setting out to examine TikTok use specifically. As a limiting consequence, our interview guide was not developed with the intention of exploring evidence of cognitive phenomena that may shape the engagement of people with eating disorders in TikTok content and the consequences of them doing so. For example, the susceptibility of individuals with AN to attentional capture by food-related stimuli [34] may increase the duration for which they view food or diet-related TikTok content, thereby increasing the likelihood of similar content being presented to them thereafter. Consuming more diet and eating disorder-related content increases users’ appearance comparison, which is in turn associated with greater ED behaviours [5, 14]. However, acknowledging that theories of attentional capture and online social comparison were not an aspect of this study’s design, we are cautious of interpreting the present data in light of them and recommend future research apply them substantively to build upon the findings of this paper.

While this paper has provided evidence of TikTok users’ lived experience of consuming ED content, the participants’ perceptions of TikTok’s content selection algorithm does not provide definitive evidence of how that algorithm functions. As such, the findings offered by this study could be usefully complemented by additional future work that records participants’ ongoing engagement with TikTok (videos watched, watch durations, reactions, etc.) and its effects upon the content with which they are subsequently provided, as well as their experiences of disordered eating. This data tracking could provide empirical evidence regarding the digital ‘rabbit holes’ that our participants identify.

That this study is the first to qualitatively consider the effect of ED content on TikTok among YP in the UK is an obvious strength. It supports existing quantitative research on TikTok algorithms and ED content (Griffith et al., 2024) while at the same time privileging the voices and experiences of those who navigate pro-ED content, TikTok algorithms, and their ED every day. The research also has the potential to enrich clinical understanding of the effects of TikTok on YP with EDs. [32] argue that there is limited understanding of the functionality of TikTok among mental health professionals; by providing an insight into the lived experience of TikTok algorithms for those with EDs, we hope to encourage clinicians to further consider the role of image-based platforms in ED prevention, treatment and care, particularly given the global reach and widespread youth use of these social media platforms.

### Recommendations

This paper provides a lived experience perspective on what echo-chambers of ED content on TikTok can be like for YP. Our findings have a number of important implications for policy, research, and practice. A key recommendation to regulatory bodies (e.g., OFCOM in the UK), and TikTok itself is to improve functions of the platform that make it safer for those who choose to engage with it – in other words, implement a harm-reduction policy (see [31]. This includes implementing effective functionality: (1) effective ‘not interested’ buttons, (2) preventing unhelpful content being displayed on the back of consuming ‘helpful’ content. Greater transparency on how the algorithm determines what content to show to users is also required. Additionally, there should be consideration generally about approaches to ED-related content and how to manage/mitigate this. Improving understanding of the effects of image-based platforms like TikTok on YP with MH issues should include involvement with researchers, clinicians, patient-carer organisations (e.g. Beat in the UK) and YP themselves in changing policies.

In terms of research implications, this paper has laid the groundwork for investigations into lived experiences of the TikTok algorithm, pro-ED content, and eating disorders. Future studies should take forward these findings by examining different populations, including boys and young men, people of colour, and people with ED diagnoses other than AN.

In terms of practice, raising awareness of the potentially detrimental effect of TikTok among YP themselves and among healthcare professionals and others working with YP will be a critical starting point. Such awareness can support the development of informed, collaborative, and supportive dialogue around social media use and its effects. For example, two of the authors of the present paper collaborated with the UK national eating disorder charity Beat to facilitate the co-production of digital resources to support YP who post or engage with ED recovery-based social media in (a) posting sensitively and (b) viewing content mindfully (see EDIFY, n.d.).

Building on these awareness-raising initiatives, interventions aiming to increase social media literacy may represent a feasible and scalable approach to tackle potentially detrimental effects of TikTok on YP. Such interventions on increasing social media literacy have been demonstrated to be effective in the prevention, early intervention and treatment of EDs [16, 18, 43].

Through guided discussions, YP can be encouraged to critically evaluate both advantages and disadvantages of contents in TikTok and their own engagement, to navigate the site with awareness and intention as well as cultivating social media literacy skills that function as protective cognitive resources (e.g., [37]. Such programmes might also be delivered via digital interventions as these offer several benefits over face-to-face interventions including cost-effectiveness and increased reach [33]. Digital interventions designed to improve media literacy and improve body image have been demonstrated to be effective with a range of participants and it is crucial for such interventions to be culturally sensitive and include girls and boys [31].

The current findings also suggest the value of embedding conversations about TikTok and social media use more broadly within existing assessment, formulation, and treatment-planning frameworks. For example, although not focused solely on TikTok, the FREED model (First Episode Rapid Early Intervention for Eating Disorders; https://freedfromed.co.uk/) includes standard assessment questions on social media use, such as which platforms are used, the amount of time spent on each, whether YP follow weight-loss or exercise accounts, and any experiences of cyber-bullying [10]. Where problematic use is identified, psychoeducational materials promoting balanced social media engagement are offered. An approach such as this can open discussions about algorithm-driven TikTok exposures that may reinforce ED behaviours and highlight potential areas for intervention and skills development during treatment. Building on these assessment and psychoeducation strategies, existing treatment models such as cognitive-behavioural therapy (CBT) could be adapted to target specific behaviours, such as compulsive scrolling, excessive engagement with the TikTok, and managing screen time, while also promoting emotional regulation, self-control, and coping mechanisms [11]. These strategies may help mitigate potential risks associated with TikTok use and foster a more mindful and intentional approach.

### Conclusion

This paper presents novel research exploring the experiences of participants with lived experience of ED and their engagement with ED content on TikTok. Using linguistic analysis of narrative extracts from YP in the UK, we found that participants’ descriptions of TikTok’s personalised algorithm suggest the perception that it may interact with, and in some cases exacerbate, ED-related behaviours and cognitions, leading users into what they describe as harmful echo-chambers of ED content. These findings support the argument that image-based platforms such as TikTok should take greater responsibility for the role of algorithmic curation in the intensification of ED symptoms and enhance mechanisms that allow users to manage the content they encounter online.

## Supplementary Information

Supplementary material 1.

## Data Availability

The transcripts of interviews generated and analysed during the current study are not publicly available as the nature of the material precluded anonymisation and participants did not consent to sharing identifiable information with third parties.

## Appendix 1

### Topic guide for interview

Please note the interviews were *semi-structured* which means we did not always ask all of the questions, or the questions in this order.

### About you

1. Tell me a bit about yourself and your experience of disordered eating. You can choose to say as much or as little as you like.
2. Through research, we have developed four lenses of people whose experience of disordered eating is underrepresented in eating disorder narratives. These are ethnic and racialised minority groups, LGBTQ+, living rurally, or experiencing resource insecurity. How – if at all – do you feel that these lenses reflect your experiences?
3. What does your circle of care look like? Who are the most important people and organisations for you?

### About social media

4) Which social media platforms do you use?
5) Talk us through how you use these platforms. If you feel comfortable, show us how you interact with the platform – what do you typically click on? How do you access your information?
6) How often do you use social media?
7) What do you use social media for?
8) What are some of the advantages and disadvantages of social media in your experience?

### About eating disorders and social media

9) Do you post about your eating disorder online?
10) If so, what aspects of your eating disorder do you post about?
11) Are the posts public or private?
12) Do you use social media to gain information about eating disorders?
13) What kind of information about eating disorders have you encountered online?
14) If you are comfortable, show us some public posts you’ve made about eating disorders. Tell us what you are trying to convey. Why did you choose to post this information?
15) What would make social media better for people with eating disorders?
16) What information online do you think would be helpful for people with eating disorders?

### Concluding questions

17) Is there anything we haven’t thought or asked about that you think is important to mention about social media and eating disorders?
18) Do you know where to go for support if needed after this interview?

## Abbreviations

CBT: Cognitive behaviour therapy
ED: Eating disorder
EDIFY: Eating disorders: delineating illness and recovery trajectories to inform personalised prevention and early intervention in young people
EOI: Expression of interest
FREED: First episode rapid early intervention for eating disorders
LGBTQ+: Lesbian, Gay, Bisexual, Transgender, Queer
OFCOM: Office of Communications
SM: Social media
SMU: Social media use
SRQR: Standards for reporting qualitative research
TA: Thematic analysis
WIEIAD: What I eat in a day
YP: Young people

## Footnotes

1 “Helpful” and “unhelpful” are participant-designated terminology; several participants referred to content such as ‘What I Eat in a Day’ as “unhelpful”, hence our decision to opt for these binary words. Nevertheless, we recognise that responses to social media content are not necessarily binary but more of a spectrum, depending on contextual factors such as mood and individual recovery stage.

2 Please note that while participant perceptions of the algorithm’s inner workings are meaningful, they should not be equated with direct evidence of algorithm design, as we did not independently verify algorithmic functioning. Consequently, any causal statements must be recognised as perceived mechanisms with clinical significance as opposed to factual descriptions of how the TikTok algorithm works.

## References

[1] Arseniev-Koehler A, Lee H, McCormick T, Moreno MA. # proana: Pro-eating disorder socialization on Twitter. J Adolesc Health. 2016;58(6):659–64. 27080731 10.1016/j.jadohealth.2016.02.012

[2] Au ES, Cosh SM. Social media and eating disorder recovery: an exploration of Instagram recovery community users and their reasons for engagement. Eat Behav. 2022;46:101651. 35760017 10.1016/j.eatbeh.2022.101651

[3] Bartel H, Downs J. Opening a New Space for Health Communication: Twitter and the Discourse of Eating Disorders in Men. In: Masculinities and Discourses of Men’s Health. Cham: Springer International Publishing; 2023. p. 77–99.

[4] Bhandari A, Bimo S. Why’s everyone on TikTok now? The algorithmized self and the future of self-making on social media. Social Media + Society. 2022;8(1):20563051221086241.

[5] Bonfanti RC, Melchiori F, Teti A, Albano G, Raffard S, Rodgers R, Coco GL. (2025). The association between social comparison in social media, body image concerns and eating disorder symptoms: A systematic review and meta-analysis. Body Image. 2025 52: 101. 10.1016/j.bodyim.2024.101841 39721448

[6] Boulianne S, Theocharis Y. Young people, digital media, and engagement: a meta-analysis of research. Soc Sci Comput Rev. 2020;38(2):111–27.

[7] Braun V, Clarke V. Using thematic analysis in psychology. Qual Res Psychol. 2006;3(2):77–101.

[8] Clarke V, Braun V. Thematic analysis. J Posit Psychol. 2017;12(3):297–8.

[9] Braun V, Clarke V. Can I use TA? Should I use TA? Should I not use TA? Comparing reflexive thematic analysis and other pattern-based qualitative analytic approaches. Couns Psychother Res. 2021;21(1):37–47.

[10] Brown A, Allen K, Beardwood J, Glennon D, Grant N, Koskina A, Mountford V, Schmidt U. The FREED care package. Guide for Clinicians; 2019.

[11] Caponnetto P, Lanzafame I, Prezzavento GC, Rawashdeh S, Moussa MA, Fakhrou A. Understanding problematic TikTok use: a systematic review of emerging diagnostic and therapeutic implications in clinical psychology. J Addict Dis. 2025;13:1–22. 10.1080/10550887.2025.2473179 40079231

[12] Davies MR, Monssen D, Sharpe H, Allen KL, Simms B, Goldsmith KA, Byford S, Lawrence V, Schmidt U. Management of fraudulent participants in online research: practical recommendations from a randomized controlled feasibility trial. Int J Eat Disord. 2024;57(6):1311–21. 37921564 10.1002/eat.24085

[13] Davis HA, Kells MR, Roske C, Holzman S, Wildes JE. A reflexive thematic analysis of# WhatIEatInADay on TikTok. Eat Behav. 2023;50:101759. 37295374 10.1016/j.eatbeh.2023.101759 PMC10526712

[14] Drivas M, Reed OS, Berndt-Goke M. # whatieatinaday: the effects of viewing food diary TikTok videos on young adults’ body image and intent to diet. Body Image. 2024;49:101712. 38636388 10.1016/j.bodyim.2024.101712

[15] Eghtesadi M, Florea A. Facebook, Instagram, Reddit and TikTok: a proposal for health authorities to integrate popular social media platforms in contingency planning amid a global pandemic outbreak. Can J Public Health. 2020;111:389–91. 32519085 10.17269/s41997-020-00343-0 PMC7282468

[16] Faccio E, Reggiani M, Rocelli M, Cipolletta S. Issues related to the use of visual social networks and perceived usefulness of social media literacy during the recovery phase: qualitative research among girls with eating disorders. J Med Internet Res. 2024;26:e53334. 38954459 10.2196/53334 PMC11252626

[17] EDIFY. Eating Disorders. Delineating illness and recovery trajectories to inform personalised prevention and early intervention in young people (EDIFY). https://edifyresearch.co.uk/10.1192/bjb.2022.83 PMC10694679 36545688

[18] Fitzsimmons-Craft EE, Krauss MJ, Costello SJ, Floyd GM, Wilfley DE, Cavazos-Rehg PA. Adolescents and young adults engaged with pro-eating disorder social media: eating disorder and comorbid psychopathology, health care utilization, treatment barriers, and opinions on harnessing technology for treatment. Eat Weight Disord-Stud Anorex Bulim Obes. 2020;25:1681–92. 10.1007/s40519-019-00808-3 PMC7195229 31679144

[19] Goh AQ, Lo NY, Davis C, Chew EC. # eatingDisorderRecovery: a qualitative content analysis of eating disorder recovery-related posts on Instagram. Eat Weight Disord-Stud Anorex Bulim Obes. 2022;18:1–1. 10.1007/s40519-021-01279-1 34537927

[20] Gray L. Exploring how and why young people use social networking sites. Educ Psychol Pract. 2018;34(2):175–94.

[21] Greene AK, Norling HN. Follow to* actually* heal binge eating”: a mixed methods textual content analysis of# BEDrecovery on TikTok. Eat Behav. 2023;50:101793. 37633221 10.1016/j.eatbeh.2023.101793

[22] Greene AK, Norling HN, Brownstone LM, Maloul EK, Roe C, Moody S. Visions of recovery: a cross-diagnostic examination of eating disorder pro-recovery communities on TikTok. J Eat Disord. 2023;11(1):109. 37400909 10.1186/s40337-023-00827-7 PMC10318659

[23] Griffiths S, Harris EA, Whitehead G, Angelopoulos F, Stone B, Grey W, Dennis S. Does TikTok contribute to eating disorders? A comparison of the TikTok algorithms belonging to individuals with eating disorders versus healthy controls. Body Image. 2024;51:101807. 39504757 10.1016/j.bodyim.2024.101807

[24] Herrick SS, Hallward L, Duncan LR. This is just how i cope: an inductive thematic analysis of eating disorder recovery content created and shared on TikTok using #EDrecovery. Int J Eat Disord. 2021;54(4):516–26. 33382136 10.1002/eat.23463

[25] Kendal S, Kirk S, Elvey R, Catchpole R, Pryjmachuk S. How a moderated online discussion forum facilitates support for young people with eating disorders. Health Expectations. 2016;20(1): 98-111. https://onlinelibrary.wiley.com/doi/full/10.1111/hex.12439} 10.1111/hex.12439 PMC5217921 26725547

[26] LENS (n.d.) Lived Experience Narratives and scenarios of eating disorders. https://futurehealthandwellbeing.org/lens

[27] Lookingbill V, Mohammadi E, Cai Y. Assessment of accuracy, user engagement, and themes of eating disorder content in social media short videos. JAMA Netw Open. 2023;6(4):e238897. 10.1001/jamanetworkopen.2023.8897. 37074713 10.1001/jamanetworkopen.2023.8897 PMC10116364

[28] Lupton D. Young people’s use of digital health technologies in the global north: narrative review. J Med Internet Res. 2021;23(1):e18286. 33427684 10.2196/18286 PMC7834940

[29] Maciejewski DF, Keijsers L, van Lier PA, Branje SJ, Meeus WH, Koot HM. Most fare well—But some do not: distinct profiles of mood variability development and their association with adjustment during adolescence. Dev Psychol. 2019;55(2):434. 30507219 10.1037/dev0000650

[30] Maloney R, Lunney L, Lennox OM, Vaughan E, Kelly C. Stigma experience of men with eating disorders: a scoping review. J Soc Care. 2024;4(1):4.

[31] Mazzeo SE, Weinstock M, Vashro TN, Henning T, Derrigo K. Mitigating harms of social media for adolescent body image and eating disorders: a review. Psychol Res Behav Manag. 2024. 10.2147/PRBM.S410600. 38978847 10.2147/PRBM.S410600 PMC11229793

[32] McCashin D, Murphy CM. Using TikTok for public and youth mental health–a systematic review and content analysis. Clin Child Psychol Psychiatry. 2023;28(1):279–306. 35689365 10.1177/13591045221106608 PMC9902978

[33] Melioli T, Bauer S, Franko DL, Moessner M, Ozer F, Chabrol H, et al. Reducing eating disorder symptoms and risk factors using the internet: a meta-analytic review. Int J Eat Disord. 2016;49(1):19–31. 26607683 10.1002/eat.22477

[34] Neimeijer RA, Roefs A, de Jong PJ. Heightened attentional capture by visual food stimuli in anorexia nervosa. J Abnorm Psychol. 2017;126(6):805. 28447804 10.1037/abn0000275

[35] Nettleton S, Burrows R, O’Malley L. The mundane realities of the everyday lay use of the internet for health, and their consequences for media convergence. Sociol Health Illn. 2005;27(7):972–92. 16313525 10.1111/j.1467-9566.2005.00466.x

[36] O'Brien BC, Harris IB, Beckman TJ, Reed DA, Cook DA. Standards for reporting qualitative research: a synthesis of recommendations. Acad Med. 2014;89(9): 1245-1251. https://www.equator-network.org/reporting-guidelines/srqr/} 10.1097/ACM.0000000000000388 24979285

[37] Paxton SJ, McLean SA, Rodgers RF. My critical filter buffers your app filter: social media literacy as a protective factor for body image. Body Image. 2022;40:158–64. 10.1016/j.bodyim.2021.12.009. 34968853 10.1016/j.bodyim.2021.12.009

[38] Peebles R, Wilson JL, Litt IF, Hardy KK, Lock JD, Mann JR, Borzekowski DL. Disordered eating in a digital age: eating behaviors, health, and quality of life in users of websites with pro-eating disorder content. J Med Internet Res. 2012;14(5):e148. 23099628 10.2196/jmir.2023 PMC3510745

[39] Radovic A, Gmelin T, Stein BD, Miller E. Depressed adolescents’ positive and negative use of social media. J Adolesc. 2017;55:5–15. 27997851 10.1016/j.adolescence.2016.12.002 PMC5485251

[40] Roehl J, Harland D. Imposter participants: overcoming methodological challenges related to balancing participant privacy with data quality when using online recruitment and data collection. Qual Rep. 2022;27(11):2469–85.

[41] Rejeb A, Rejeb K, Appolloni A, Treiblmaier H, Iranmanesh M. Mapping the scholarly landscape of TikTok (Douyin): a bibliometric exploration of research topics and trends. Digital Business. 2024;22:100075.

[42] Rouleau CR, von Ranson KM. Potential risks of pro-eating disorder websites. Clin Psychol Rev. 2011;31(4):525–31. 21272967 10.1016/j.cpr.2010.12.005

[43] Schmidt U, Brown A, McClelland J, Glennon D, Mountford VA. Will a comprehensive, person-centered, team-based early intervention approach to first episode illness improve outcomes in eating disorders? Int J Eat Disord. 2016. 10.1002/eat.22519. 27084796 10.1002/eat.22519

[44] Sefcik JS, Hathaway Z, DiMaria-Ghalili RA. When snowball sampling leads to an avalanche of fraudulent participants in qualitative research. Int J Older People Nurs. 2023;18(6):e12572. 37632269 10.1111/opn.12572 PMC10843676

[45] Sharma A, Vidal C. A scoping literature review of the associations between highly visual social media use and eating disorders and disordered eating: a changing landscape. J Eat Disord. 2023;11(1):170. 37752611 10.1186/s40337-023-00898-6 PMC10521472

[46] Steakley-Freeman DM, Jarvis-Creasey ZL, Wesselmann ED. What’s eating the internet? Content and perceived harm of pro-eating disorder websites. Eat Behav. 2015;19:139–43. 26363674 10.1016/j.eatbeh.2015.08.003

[47] Steinberg L. Risk taking in adolescence: new perspectives from brain and behavioral science. Curr Dir Psychol Sci. 2007;16(2):55–9.

[48] Valkenburg PM, Meier A, Beyens I. Social media use and its impact on adolescent mental health: an umbrella review of the evidence. Curr Opin Psychol. 2022;44:58–68. 34563980 10.1016/j.copsyc.2021.08.017

[49] Wall Street Journal. Inside tiktok’s algorithm: A WSJ video investigation. Wall Street J. 2021.

[50] Wang V, Edwards S. Strangers are friends I haven’t met yet: a positive approach to young people’s use of social media. J Youth Stud. 2016;19(9):1204–19.

[51] Zheng DX, Mulligan KM, Scott JF. TikTok and dermatology: an opportunity for public health engagement. J Am Acad Dermatol. 2021;85(1):e25–6. 33639245 10.1016/j.jaad.2021.02.050

