# Supplementary material for: ‘Falling down the rabbit hole’: a thematic analysis of young people’s views on TikTok algorithms and eating disorder content
Source: J Eat Disord. 2025 Dec 20;14:27. doi: 10.1186/s40337-025-01505-6 (PMC12831324; doi:10.1186/s40337-025-01505-6)
Supplement: Supplementary file 1 — Supplementary material 1. [file 40337_2025_1505_MOESM1_ESM.docx]

**Supplementary information 1 –** Standards for Reporting Qualitative Research Checklist (SRQR)

| No | Topic | Item | Page number |
| --- | --- | --- | --- |
|  | Title and abstract |  |  |
| S1 | Title | Concise description of the nature and topic of the study identifying the study as qualitative or indicating the approach or data collection methods. | p. 1 |
| S2 | Abstract | Summary of key elements of the study using the abstract format of the intended publication. | pp. 1–2 |
|  | Introduction |  |  |
| S3 | Problem Formulation | Description and significance of the problem/phenomenon studied. | pp. 2–5 |
| S4 | Purpose or research question | Purpose of the study and specific objectives or questions. | p. 5 |
|  | Methods |  |  |
| S5 | Qualitative approach and research paradigm | Qualitative approach and guiding theory if appropriate; identifying the research paradigm is also recommended. | pp. 8–9 |
| S6 | Researcher characteristics and reflexivity | Researcher’s characteristics that may influence the research, including personal attributes, qualifications/experience, relationship with participants, assumptions, potential or actual interaction between researchers’ characteristics and the research questions, approach, methods, results, and/or transferability. | pp. 9–10 |
| S7 | Context | Setting/site and salient contextual factors; rationale. | p. 6 |
| S8 | Sampling strategy | How and why research participants, documents, or events were selected; criteria for deciding when no further sampling was necessary (e.g. sampling saturation), rationale. | pp. 5–6; pp. 7–8 (Table 1) |
| S9 | Ethical issues pertaining to human subjects | Documentation of approval by an appropriate ethics review board and participant consent, or explanation for lack therof; other confidentiality and data security issues. | p. 5 |
| S10 | Data collection methods | Types of data collected; details of data collection procedures including start and stop dates of data collection and analysis, iterative process, triangulation of sources/methods, and modification of procedures in response to evolving study findings. | Mention of iterative process and triangulation: p. 9 |
| S11 | Data collection instruments and technologies | Description of instruments (e.g. interview guides, questionnaires) and devices used for data collection; if/how the instrument(s) changed over the course of the study. | p. 6 |
| S12 | Units of study | Number and relevant characteristics of participants, documents, or events included in the study; level of participation (could be reported in results). | p. 7–8, Table 1 |
| S13 | Data processing | Methods for processing data prior to and during analysis; including transcription, data entry, data management and security, verification of data integrity, data coding, and anonymisation/deidentification of excerpts. | p. 9 (transcription, data entry, coding); p. 6 (data management and security) |
| S14 | Data analysis | Process by which inferences, themes etc. were identified and developed, including the researchers involved in data analysis; usually references a specific paradigm or approach. | p. 9 |
| S15 | Techniques to enhance trustworthiness | Techniques to enhance trustworthiness and credibility of data analysis. | achieved through iterative analytical process as explained on p. 9 |
|  | Results/findings |  |  |
| S16 | Synthesis and interpretation | Main findings. | pp. 10–18 |
| S17 | Links to empirical data | Evidence to substantiate analytical findings. | pp. 10–18 |
|  | Discussion |  |  |
| S18 | Integration with prior work, implications, transferability and contribution to the field | Short summary of main findings; explanation of how findings and conclusions connect to, support, elaborate on or challenge conclusions of earlier scholarship; discussion of scope of application/generalisability; identification of unique contributions to scholarship in a discipline or field. | pp. 18–21 |
| S19 | Limitations | Trustworthiness and limitations of findings. | pp. 21–22 |
|  | Other |  |  |
| S20 | Conflicts of interest | Potential sources of influence or perceived influence on study conduct and conclusions; how these were managed. | p. 31 |
| S21 | Funding | Sources of funding and other support; role of funders in data collection, interpretation, and reporting. | p. 31 |
